# ALADDIN: Docking Approach Augmented by Machine Learning for Protein Structure Selection Yields Superior Virtual Screening Performance

**DOI:** 10.1002/minf.201900103

**Published:** 2019-11-08

**Authors:** Ningning Fan, Christoph A. Bauer, Conrad Stork, Christina de Bruyn Kops, Johannes Kirchmair

**Affiliations:** ^1^ Universität Hamburg, Faculty of Mathematics, Informatics and Natural Sciences, Department of Informatics Center for Bioinformatics 20146 Hamburg Germany; ^2^ University of Bergen Department of Chemistry N-5020 Bergen Norway; ^3^ University of Bergen Computational Biology Unit (CBU) N-5020 Bergen Norway

**Keywords:** virtual screening, ensemble docking, machine learning, structure selection, similarity-based docking

## Abstract

Protein flexibility and solvation pose major challenges to docking algorithms and scoring functions. One established strategy for addressing these challenges is to use multiple protein conformations for docking (all‐against‐all ensemble docking). Recent studies have shown that the performance of ensemble docking can be improved by selecting the most relevant protein structures for docking. In search for a robust approach to protein structure selection, we have come up with an integrated mAchine Learning AnD DockINg approach (ALADDIN). ALADDIN employs a battery of random forest classifiers to select, individually for each compound of interest, from an ensemble of protein structures, the single most suitable protein structure for docking. ALADDIN outperformed the best single‐structure docking runs, ensemble docking and a similarity‐based docking approach on three out of four investigated targets, with up to 0.15, 0.11 and 0.16 higher area under the receiver operating characteristic curve (AUC) values, respectively. Only in the case of cytochrome P450 3A4, ALADDIN, like any of the other tested approaches, failed to obtain decent performance. ALADDIN can be particularly useful for structure‐based virtual screening of malleable proteins, including kinases, some viral enzymes and anti‐targets.

## Graphical abstract

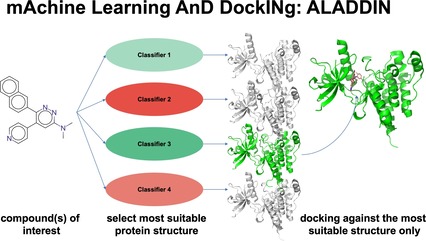

## 1 Introduction

Ligand docking is one of the most widely applied computational approaches in drug discovery.^1^, ^2^, ^3^ Modern docking algorithms and scoring functions are powerful tools for predicting the likely binding pose of small molecules.^4^ They also have a strong track record in virtual screening.^5^ The largest docking study reported to date includes the virtual screening of a total of 170 million make‐on‐demand compounds against AmpC β‐lactamase and the D_4_ dopamine receptor, as a result of which several novel and, in part, highly potent inhibitors of these proteins were identified.^6^ Despite these successes, the ability of scoring functions to estimate in particular absolute ligand binding affinities remains clearly limited,^7^, ^8^ which is related to the inadequate consideration of protein flexibility,^9^, ^10^ solvation effects,^10^, ^11^ and entropy.^12^ The computational costs involved in sampling the relevant conformational states of biomacromolecules are often prohibitive to the consideration of protein flexibility and solvation in docking, in particular in the context of virtual screening. One of the most widely applied strategies to mitigate this problem is to generate ensembles of representative (and generally static) target structures for docking.^13^, ^14^ In this so‐called (all‐against‐all) ensemble docking approach, ligands of interest are individually docked against each of the ensemble structures, and the predictions assessed according to user‐defined scoring schemes.^15^, ^16^

Ensembles are commonly compiled from sets of X‐ray structures,^17^, ^18^ homology models,^19^ frames extracted from molecular dynamics (MD) trajectories,^13^, ^20^ or combinations thereof. Several studies have demonstrated the potential of ensemble docking to improve early enrichment, pose prediction, and coverage of the bioactive chemical space.^18^, ^21^, ^22^, ^23^ The benefit of ensemble docking over single‐structure docking can further be improved by methods allowing the identification of the most suitable ensembles for docking. For instance, Rao et al.^24^ found, by the example of p38 MAP kinase, that small ensembles of protein structures yielding high docking scores for the top‐ranked ligands are likely to also yield high early enrichment. Korb et al.^22^ showed that the performance of ensemble docking is determined, among other factors, by the structural similarity between the compound(s) of interest and the co‐crystallized ligands: structures of proteins based on co‐crystals with structurally related ligands promise higher docking success rates. Such findings have also been made in earlier studies.^25^, ^26^ Another study found that reduced ensembles of just three to five protein structures could be generated by taking into account the virtual screening performance on small data sets of known active and inactive compounds. This approach was found to improve both the efficiency and performance of ensemble docking.^27^ Swift et al.^23^ explored three knowledge‐based strategies to generate ensembles of protein structures yielding maximum virtual screening performance. More recently, an approach for the pre‐selection of protein structures for docking (“ProSelection”) was introduced, which identifies protein structures as “strong selectors” or “weak selectors” based on the distribution of docking scores among the inactive and active compounds.^28^

In this work, we develop and test a new docking strategy that integrates machine learning to select, individually for each compound of interest, from an ensemble of protein structures, the single most suitable protein structure for docking. We refer to this method as the integrated approach for mAchine Learning AnD DockINg (ALADDIN). ALADDIN has the potential to not only yield higher docking performance than established (ensemble) docking protocols but also to boost computational efficiency.

## 2 Methods

### 2.1 ALADDIN

**Figure 1:**
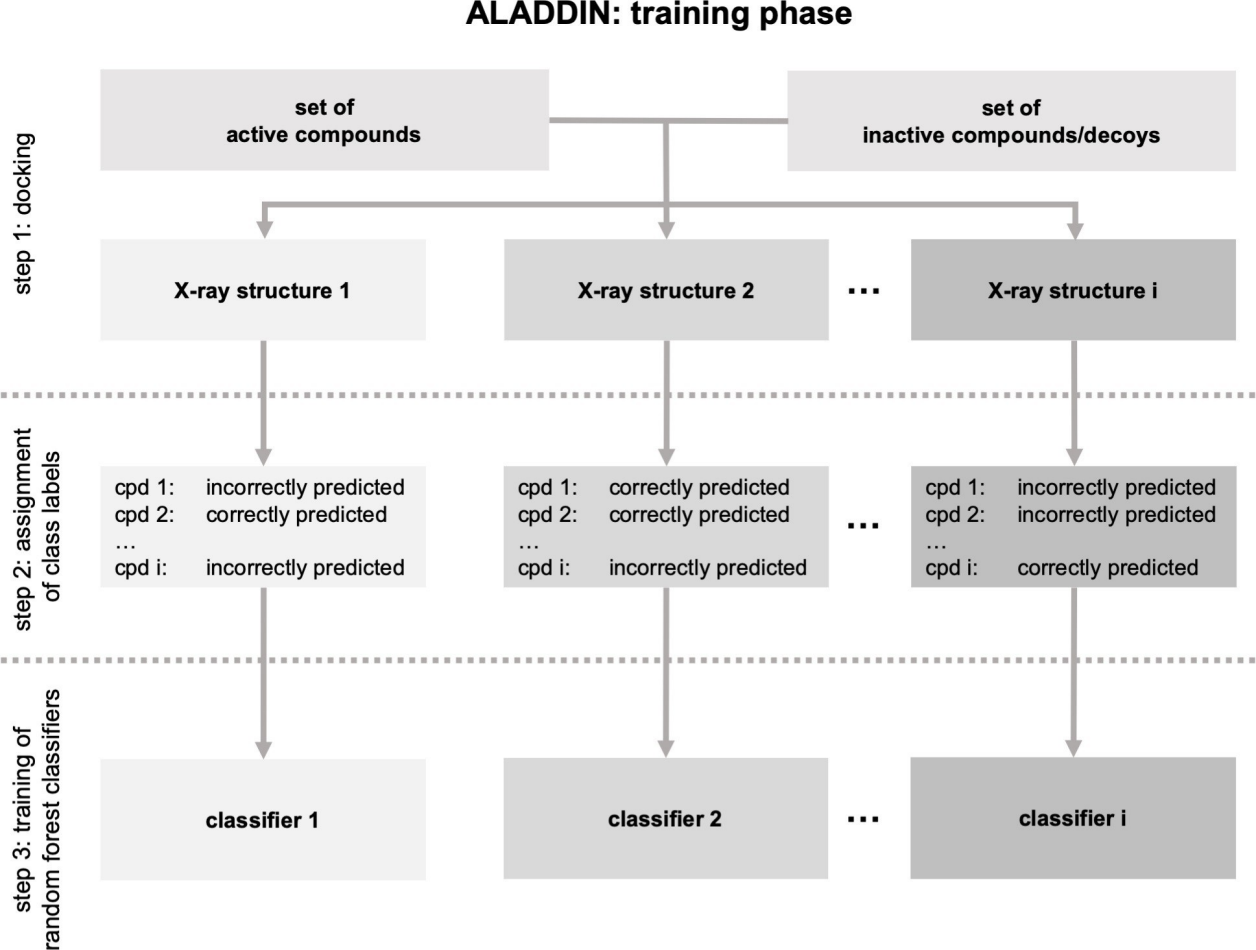
Overview of the training phase of ALADDIN.

The training phase of ALADDIN consists of the following steps that are executed in sequence (Figure 1):

1. A set of known ligands and inactive compounds (or decoys) is docked against a set of protein structures (this part corresponds to the classic all‐against‐all ensemble docking approach).
2. Individually for all (rank‐ordered) hit lists obtained in step 1, compounds are assigned a binary label, indicating whether or not they were correctly predicted by the docking approach. The value “correctly predicted” is assigned to any actives that obtained “high ranks” during docking (i. e., low GlideScore values, since GlideScore approximates binding free energies) and to any decoys that obtained “low ranks”. Likewise, the value “incorrectly predicted” is assigned to any actives with “low ranks” assigned, and to any decoys with “high ranks” assigned. Thereby, “high ranks” are defined as any ranks better or equal to n, where n is the number of active compounds in the data set, and all others were defined as “low ranks”.
3. Individually for all hit lists obtained in step 1 (and hence, individually for all protein structures), a binary random forest classifier is trained that aims to learn which compounds, based on the binary class labels assigned in step 2, are correctly predicted by docking as active or inactive and which ones are not.

**Figure 2:**
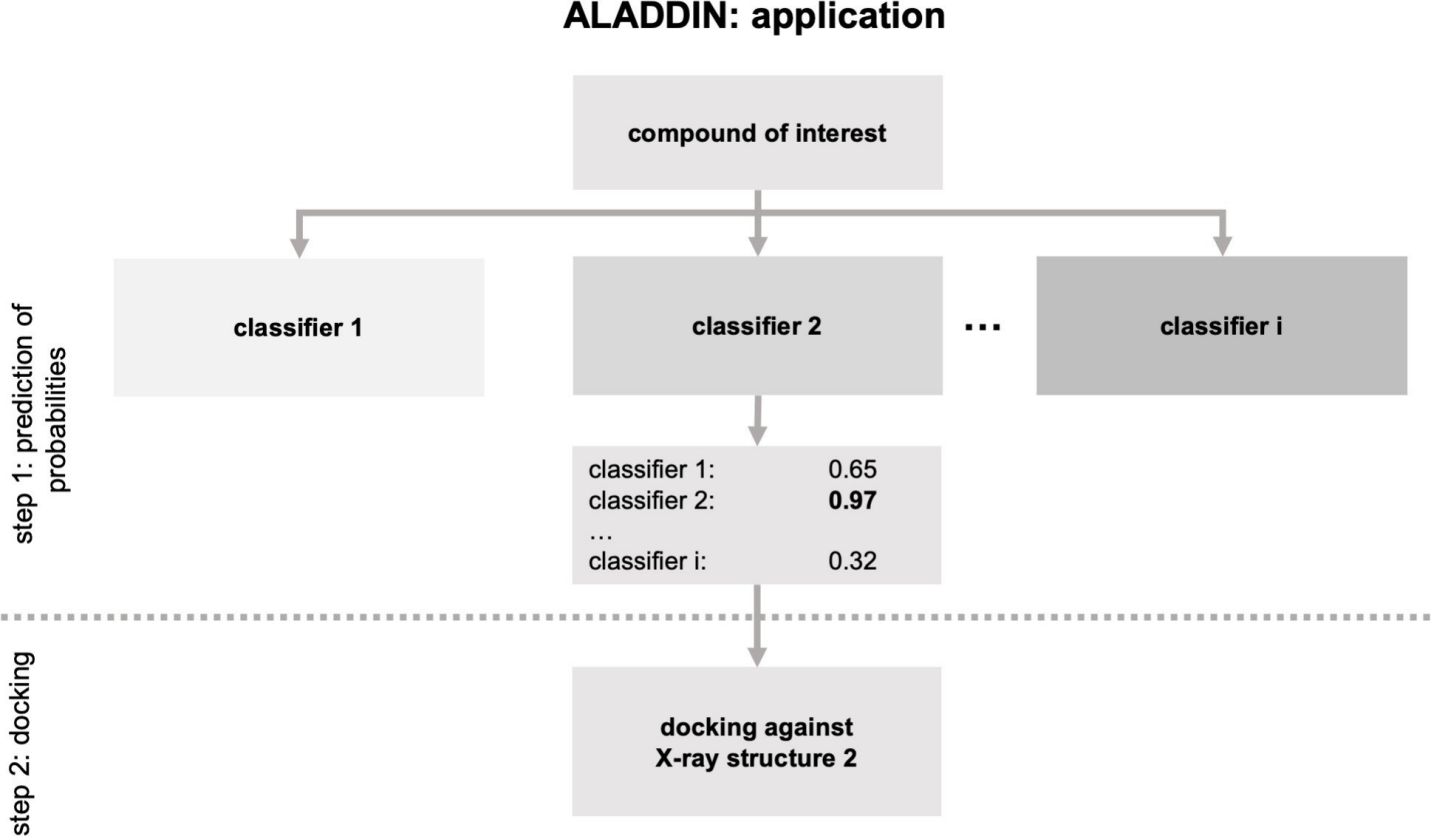
Overview of the application of ALADDIN to compounds of interest.

After completion of the training phase, the battery of binary random forest classifiers (one for each protein structure) is used for identifying the single most suitable protein structure for docking (Figure 2):

1. A compound of interest is presented to each of the classifiers to obtain probability values.
2. The compound of interest is docked against the protein structure for which the highest probability value was obtained from any of the classifiers.

The docking pose and score resulting from this single docking process is the outcome of ALADDIN.

### 2.2 Data Preparation

**Protein Structural Data**. For each protein studied in this work (i. e. human vascular endothelial growth factor receptor 2, VEGFR2; human MAP kinase p38 alpha, p38α MAPK; human glucocorticoid receptor, GCR; human cytochrome P450 3A4, CYP3A4), all holo X‐ray structures (identified by UniProtKB accession numbers) with resolution better than 2.5 Å were downloaded from the Protein Data Bank (PDB; Table 1). For oligomers, all chains with at least one co‐crystallized ligand present were treated as individual structures. In the case of p38α MAPK, because of the large number of available protein structures, an ensemble of representative protein structures was generated with SIENA^29^ (all settings default). All selected structures were prepared using the Protein Preparation Wizard^30^ within Maestro.^31^ After preprocessing with default settings, missing atoms of amino acid side chains were added with Prime.^32^ All water molecules were removed from the protein structure. Next, restrained minimization of the protein structures was performed with the OPLS3e force field^33^ and a default convergence RMSD tolerance of 0.3 Å compared to the input structures.

**Table 1:** Overview of Structures Compiled from the PDB.

Target	Protein accession ID	No. of PDB entries		No. of target structures selected for docking^b^	Retrieval date
		retrieved	valid^a^		
VEGFR2	P35968	35	32	38	June 12, 2019
p38α MAPK	Q16539	199	161	30	Nov 15, 2018
GCR	P04150	37	20	30	June 12, 2019
CYP3A4	P08684	28	21	25	June 12, 2019

^a^ Valid structures are any structures with a ligand observed in the binding site occupied by the representative structure deposited in the DUD‐E and with a resolution better than 2.5 Å. ^b^ Number of structures of individual protein chains selected for docking. One PDB entry may be represented by more than one structure. In the case of p38α MAPK, because of the high number of valid protein structures, SIENA was employed to generate a representative ensemble of protein structures.

**Small‐Molecule Data**. For each of the four proteins, the complete set of active compounds and decoys was retrieved from the Directory of Useful Decoys, Enhanced (DUD‐E)^34^ in SMILES format (Table 2). The structures were prepared using LigPrep^35^ within Maestro.^31^ For each molecule, a single representation of the most likely ionization and tautomeric state at pH 7.0 was calculated with Epik.^36^ Subsequently, the energy of the generated conformer was minimized with the OPLS3e force field with default parameters.

**Table 2:** Sizes of the Small‐Molecule Data Sets and Subsets Prior and After Preprocessing.

Target	No. of compounds											
	Prior to pre‐ processing^a^		After pre‐ processing^b^		Training set		Test set^c^		Test subset 1^d^		Test subset 2^e^	
	Actives	Decoys	Actives	Decoys	Actives	Decoys	Actives	Decoys	Actives	Decoys	Actives	Decoys
VEGFR2	2320	24950	2320	24937	1853	19953	467	4984	225	3925	68	3269
p38α MAPK	2218	35850	2218	35833	1804	28637	414	7196	229	5822	75	4761
GCR	992	15000	992	14994	800	11989	192	3005	77	2573	21	2196
CYP3A4	303	11800	303	11797	240	9440	63	2357	35	2114	19	1888

^a^ Complete data sets downloaded from the DUD‐E database in SMILES format. ^b^ For a small number of compounds, no 3D conformation could be generated with LigPrep. ^c^ Consisting of 20 % of the respective DUD‐E subset This is the complete test set (i. e., 20 % of the respective DUD‐E dataset). ^d^ Subset of the test set, consisting only of molecules having a maximum Tanimoto coefficient (Morgan2 fingerprints with 1024 bits) of 0.8 with any of the compounds present in the training data. ^e^ Subset of the test set, consisting only of molecules having a maximum Tanimoto coefficient (Morgan2 fingerprints with 1024 bits) of 0.7 with any of the compounds present in the training data.

**Docking**. In preparation for docking, Glide receptor grids, centered on the co‐crystallized ligand of the individual protein structures, were generated. The option “dock ligands similar in size to the workspace ligand” was selected to define the size of the receptor box. In the case of the presence of alternative ligand conformations, the first conformation recorded in the PDB file was selected to define the grid.

Docking was conducted with the Glide Standard Precision (Glide SP) algorithm^37^ with default settings (i. e. enabled sampling of nitrogen inversions; enabled sampling of ring conformations with an energy window of 2.5 kcal/mol; enabled bias sampling of amides only with penalization of nonplanar conformations). For ligands represented by more than one molecular structure (e. g. in the case of tautomers or protomers), the highest GlideScore obtained with any representation of a molecule was considered.

**Machine Learning**. Prior to the training of random forest classifiers, each of the DUD‐E actives and decoys sets was split into a training and a test set with a ratio of 80 : 20 (Table 2). For each individual protein structure, a random forest classifier was trained with scikit‐learn^38^ on all compounds of the respective training set. The class labels, assigned according to the method described above (“correctly predicted”, “incorrectly predicted”), served as the dependent variable. The *class_weight* parameter for the random forest classifier was set to “balanced” (i. e. weights adjusted to be inversely proportional to the class frequencies in the training data). The optimum setup for training random forest classifiers was determined by a grid search within a 10‐fold cross‐validation framework, as part of which a variety of combinations of hyperparameters and molecular descriptors were explored (Table 3). Thereby, the Matthews correlation coefficient (MCC), averaged across all folds, served as performance measure.

**Table 3:** Descriptors, Labeling Schemes and Random Forest Hyperparameters Explored in this Work.

Components	Values
Descriptors	MACCS keys^a^, Morgan fingerprints^a^, MOE 2D descriptors^b^
Number of estimators^c^	50, 100, 500
Maximum number of features^d^	“sqrt”, 0.2, 0.4, 0.6, 0.8, “None”

^a^ Implemented in RDKit.^39^ ^b^ (All) 206 2D descriptors implemented in MOE.^40^ ^c^ Number of trees in the forest. ^**d**^ Maximum Number of features considered for identifying the best split.

## 3 Results

ALADDIN was tested on four representative human proteins of pharmaceutical relevance for which we retrieved sets of known ligands and decoys from the DUD‐E:

- VEGFR2, a principle responder to vascular endothelial growth factor signal and the major signal transducer for angiogenesis.^41^, ^42^
- p38α MAPK, which mediates cellular responses to injurious stress and immune signaling and regulates tumorigenesis.^43^, ^44^
- GCR, a nuclear receptor controlling the transcription within networks comprising thousands of genes and dominating in various fields of development, metabolism, stress response, inflammation and other organismal processes.^45^
- CYP3A4, a member of cytochrome P450 family which metabolizes a large variety of xenobiotics and endogenous compounds.^46^

VEGFR2 and p38α MAPK were selected as representative protein kinases with differing amounts of X‐ray structural data available. For VEGFR2, we identified 32 valid PDB structures, corresponding to 38 protein chains (“target structures”) that were selected for the docking experiments (Table 1; see Methods). In the case of p38α MAPK, a much higher number of structures are available from the PDB (199 structures). For this reason, we employed SIENA, an automated approach for the generation of representative protein structure ensembles, for the reduction of target conformations used for docking. Specifically, we used a structure of a heterobicyclic inhibitor bound to p38α MAPK (PDB ID: 2QD9; serving as the reference structure for DUD‐E) as query for the generation of an ensemble of 30 representative structures of the human p38α MAPK.

GCR is known as a challenging target for structure‐based virtual screening because its ligand binding pocket is flexible and highly hydrophobic. The AUC obtained by a docking approach on GCR was the second lowest across all targets included in the DUD‐E.^34^ CYP3A4 is a further target known to pose significant challenges to structure‐based virtual screening. The enzyme is highly malleable and has a large, hydrophobic binding pocket that lacks clear pharmacophoric requirements for ligand binding. For GCR and CYP3A4, the protein structure selection procedure resulted in 30 and 25 target structures, respectively. Detailed information on the structures selected for the individual targets is provided in Tables S1 to S4.

### 3.1 Performance of Single‐Structure Docking

**Figure 3:**
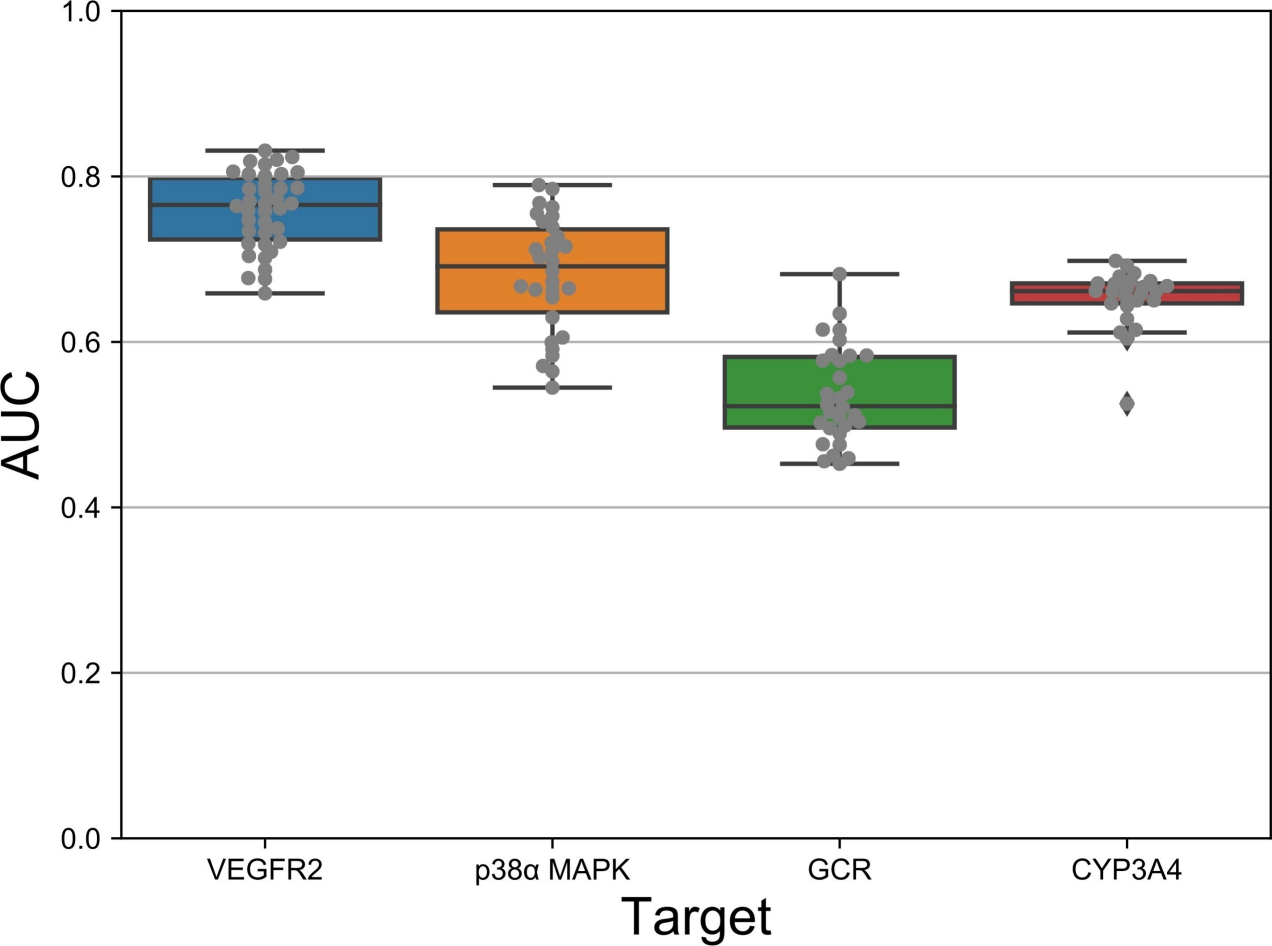
Spread of AUC values for single‐structure docking on the test set. The mean AUC values for VEGFR2, p38α MAPK, GCR and CYP3A4 were 0.76 (σ=0.05), 0.68 (σ=0.07), 0.54 (σ=0.06) and 0.65 (σ=0.04), respectively. The outlier observed among the CYP3A4 structures is 6MA6, a co‐crystal with metyrapone bound. Metyrapone is a small inhibitor of CYP3A4; its molecular weight is just 226.27 g/mol.

**Figure 4:**
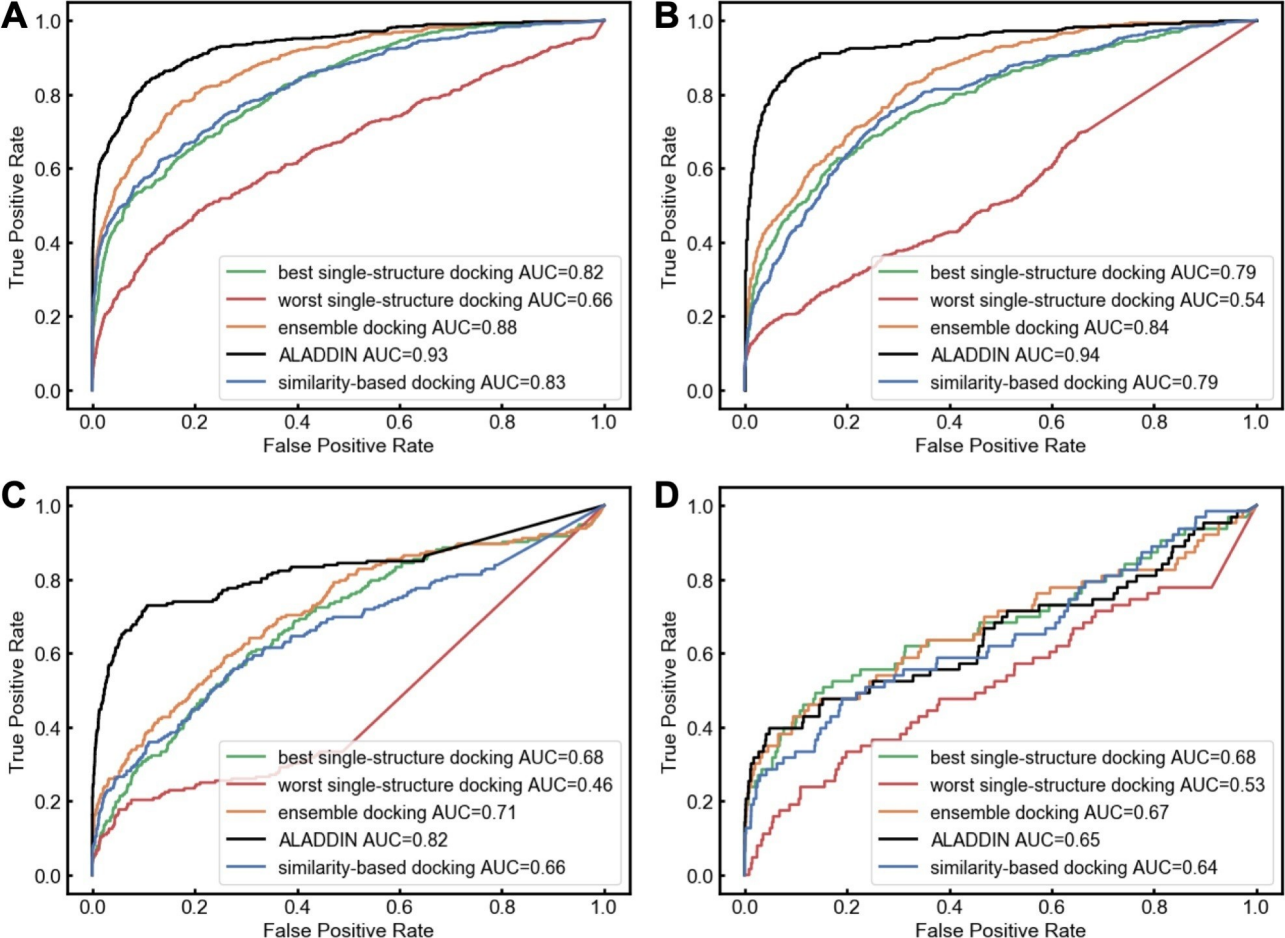
ROC curves and AUC values obtained on the test sets of the four targets: (A) VEGFR2, (B) p38ɑ MAPK, (C) GCR, (D) CYP3A4. “best single‐structure” and “worst single‐structure” denote the protein structures for which the best and worst performances were obtained on the full data set, respectively (Tables S1 to S4).

**Figure 5:**
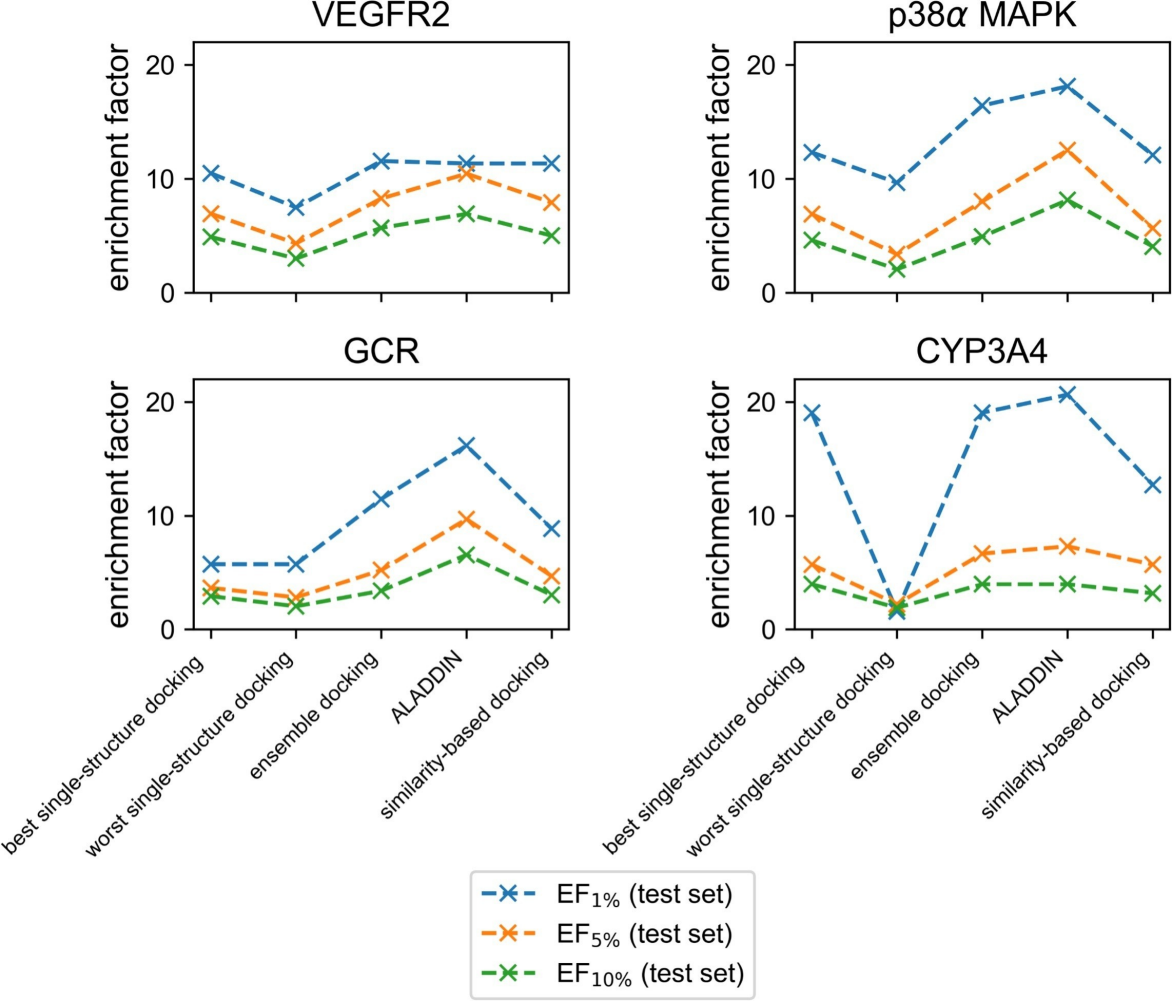
Enrichment factors obtained for the test set. The dashed lines are merely a guide to the eye. Note that enrichment factors are dependent on the composition of data sets. Enrichment factors obtained for the individual targets should therefore not be used for direct comparison.

In order to set reference points for comparing the performance of different docking strategies, we explored the range of AUC values obtained by single‐structure docking for the identical sets of protein structures that will also be used for evaluating ensemble docking and ALADDIN. Unless stated otherwise, all values presented in this work refer to the performance on the test set. Single‐structure docking obtained mean AUC values of 0.76, 0.68, 0.54 and 0.65 for VEGFR2, p38α MAPK, GCR and CYP3A4, respectively (Figure 3). For the individual targets some substantial differences in AUC values and early enrichment were observed between the best and the worst docking run (Figure 4 and 5). For example, for p38α MAPK the best ROC curve (AUC 0.79) indicates decent performance of the docking algorithm whereas the worst ROC curve (AUC 0.54) indicates a performance that is close to random selection (Figures 4).

In several structures used in this work, the side chains of some amino acids are missing. We modeled these with Prime. However, some structures have larger parts unresolved, in particular flexible loops (such as the DFG loop in the case of VEGFR2). We tried to model also these parts with Prime. However, in several virtual screening experiments with GLIDE we found the structures of modeled loops to be inaccurate, for which reason we decided to refrain from modeling larger unresolved protein parts and go ahead with the original, preprocessed structures.

### 3.2 Performance of Ensemble Docking

For three out of the four targets investigated in this work, (all‐against‐all) ensemble docking outperformed single‐structure docking on the test set. AUC values were between 0.12 (VEGFR2) and 0.17 (GCR) higher than the average AUC values obtained by single‐structure docking (Figure 3), and also between 0.03 (GCR) and 0.06 (VEGFR2) higher than for the best single‐structure docking runs (Figure 4). For CYP3A4, no gain in performance of ensemble docking over single‐structure docking was observed. As shown in Figure 5, early enrichment follows the trends observed for AUC values. Compared by their EF_5%_ values, ensemble docking matches the performance of the best single‐structure run.

### 3.3 Performance of ALADDIN

Prior to discussing the performance of the integrated (ALADDIN) approach, we briefly report on the performance of the individual machine learning models (i. e. their ability to predict which compounds will be correctly docked).

**Performance of the Machine Learning Models**. The best classifiers, optimized during a grid search within the framework of 10‐fold cross‐validation (see Methods for details), obtained MCC values (averaged over all folds and models) between 0.51 (CYP3A4) and 0.62 (GCR), with low standard deviations (Table 4). The selected components and parameters were consistent across the four targets (Table 4). Morgan fingerprints performed best among the three sets of descriptors investigated. Different radii (2, 3, 4) and bit lengths (1024, 2048) for Morgan fingerprints were explored at the example of VEGFR2. Since no substantial changes in performance were observed (Table S5), a radius of 2 and a bit length of 1024 were selected for all subsequent experiments (Tables S6–S8). Chance correlation was excluded by a Y‐scrambling test (the averaged MCC values were 0.00 for all targets). The final models were trained on the full training sets, this time balanced with the Synthetic Minority Over‐sampling Technique, SMOTE,^47^ with the optimum modeling setup identified during the grid search.

**Table 4:** Overview of the Selected Modeling Setup and the Performance of the Best Models on the Training Set.

Components	VEGFR2	p38α MAPK	GCR	CYP3A4
Descriptors	Morgan2 fingerprints with 1024 bits			
Number of estimators	500			
Maximum number of features	sqrt			
MCC averaged over all folds and models	0.58	0.60	0.62	0.51
Standard deviation (σ)	0.02	0.03	0.02	0.02

**Performance of the Integrated Approach**. ALADDIN reached superior virtual screening performance over the single‐structure and ensemble docking approaches for VEGFR2, p38α MAPK, and GCR. One of the strongest increases in performance on the test set was observed for GCR, for which ALADDIN reached an AUC of 0.82 whereas ensemble docking and the best single‐structure docking run yielded AUC values of only 0.71 and 0.68, respectively (Figure 4). In the case of VEGFR2, ALADDIN obtained an AUC of 0.93, which is 0.05 higher than the AUC obtained by the ensemble approach and 0.11 higher than the AUC obtained by the best single‐structure docking run. Similar results were obtained for p38α MAPK, where ALADDIN yielded an AUC of 0.94, ensemble docking an AUC of 0.84, and the best single‐structure run an AUC of 0.79. These observations hold true also for enrichment factors, where ALADDIN obtained higher EF_5%_ and EF_10%_ values for VEGFR2, p38α MAPK, and GCR than any of the other docking approaches (Figure 5 and Tables S9 to S11). For example, in the case of p38α MAPK, the EF_5%_ and EF_10%_ were 12.51 and 8.14 for ALADDIN, whereas they were only 8.02 and 4.93 for ensemble docking, respectively. Only in the case of CYP3A4, ALADDIN failed, like any of the tested established docking approaches, to reach decent performance (AUC 0.65). The AUC values obtained by any of the investigated docking approaches were between 0.53 and 0.68.

In order to test the robustness of ALADDIN, the method was also tested on subsets of the test sets that are composed of molecules that are less closely related to the structures represented by the training data. More specifically, (for each target) subset 1 is composed of molecules with a maximum Tanimoto coefficient (Morgan2 fingerprints with 1024 bits) of 0.8 calculated for any pair of training and test set compounds; subset 2 was compiled in the same fashion but with a cutoff of 0.7. Also on these subsets, ALADDIN outperformed all other investigated docking approaches. For subset 1, the gain in AUC of ALADDIN over ensemble docking was between 0.04 (VEGFR2) and 0.13 (GCR); for subset 2 it was between 0.02 (VEGFR2) and 0.17 (GCR). The same trends were observed for the enrichment factors (Tables S9–S11).

### 3.4 Performance of Similarity‐based Docking

We have shown that ALADDIN outperforms other docking approaches on three out of four targets, the exception being CYP3A4, where all tested approaches fail to obtain decent early enrichment. What is yet to be tested is whether the random forest‐based ALADDIN brings added value over a simple similarity‐based docking approach akin to that of Korb et al.,^22^ which can be considered a baseline experiment. In this approach, compounds of interest are individually docked against the target structure that is derived from the complex with the most similar bound ligand (similarity defined as Tanimoto coefficient calculated on Morgan2 fingerprints with a length of 1024 bits). As apparent from Figure 4, ALADDIN performs substantially better on the test sets than the similarity‐based docking approach, with AUC values 0.10, 0.15 and 0.16 higher for VEGFR2, p38α MAPK, and GCR, respectively. Again, these observations are consistent with those made for the early enrichment rates (Tables S9 to S11). Unsurprisingly, also the similarity‐based docking approach fails to yield decent screening performance for CYP3A4.

As a final note on the comparative method assessment, we hold that the training set (Figure S1) and test set performances are consistent throughout for all approaches in both AUC and enrichment factor metrics.

### 3.5 In‐Depth Analysis of the ALADDIN Model Behavior

**Figure 6:**
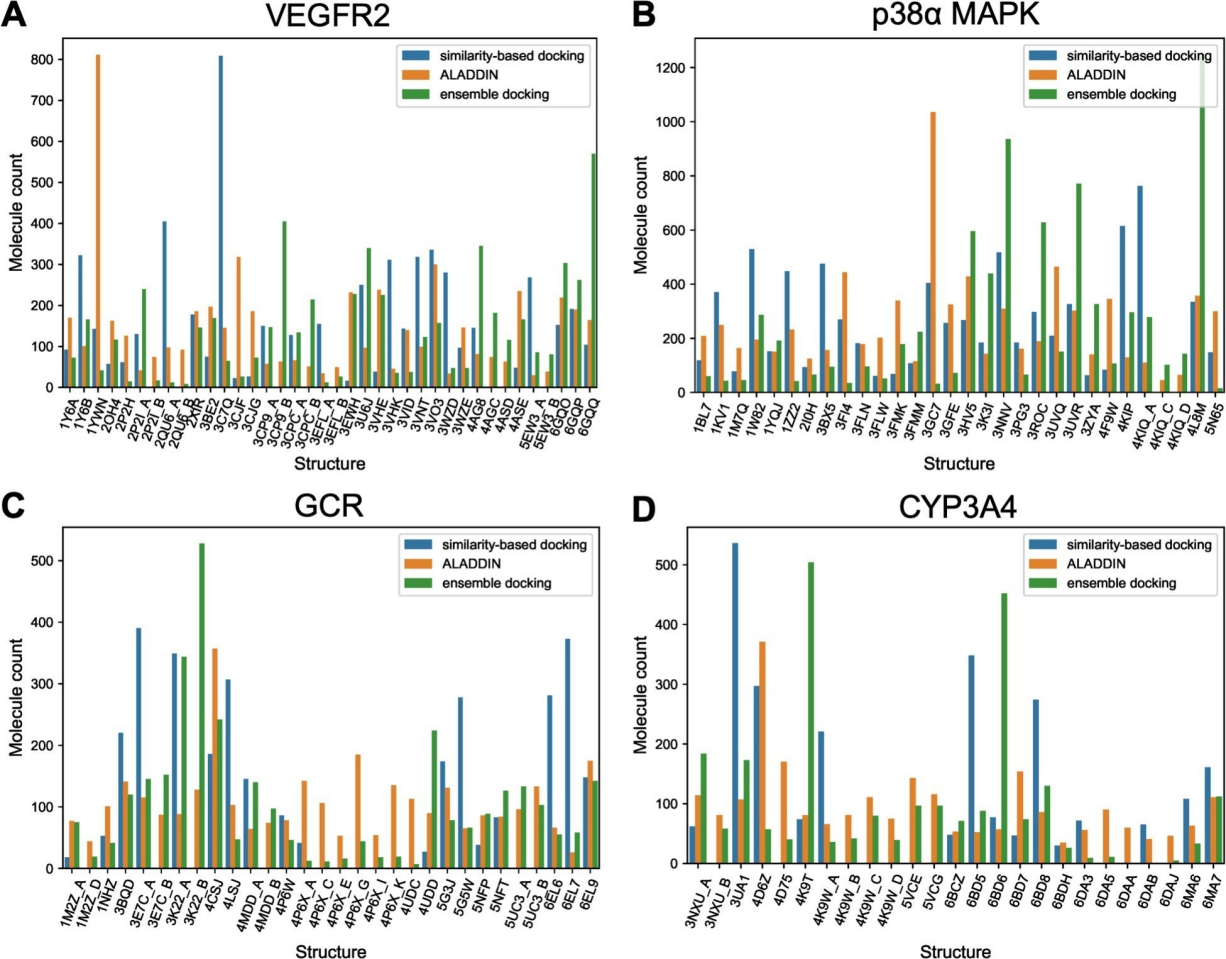
Plot reporting the number of molecules of the test sets for which a specific structure was selected for docking: (A) VEGFR2, (B) p38α MAPK, (C) GCR and (D) CYP3A4.

In order to obtain a better understanding of ALADDIN, we investigated its behavior with respect to the selection of protein structures for docking. From Figure 6 it is apparent that ALADDIN has a clear preference for a single protein structure, and this is consistent across all four targets. The ensemble docking and similarity‐based docking approaches also show preferences for individual protein structures but overall their selection of structures is more balanced.

**Figure 7:**
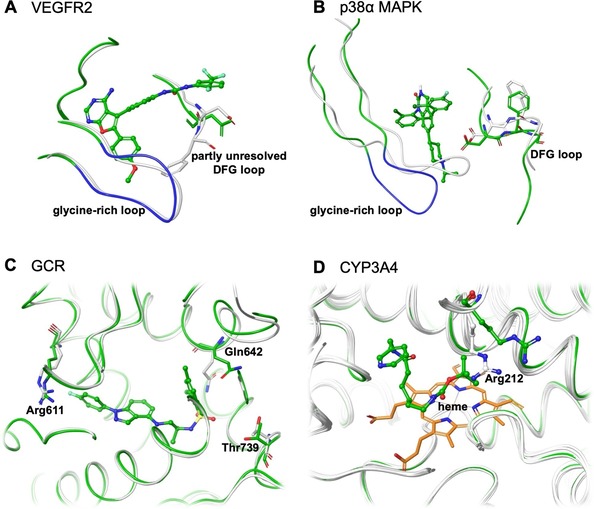
Comparison of the structure preferred by ALADDIN (green) with other selected structures (grey): (A) VEGFR2 (1YWN in green with glycine‐rich loop in blue, preferred by ALADDIN; 3C7Q in grey, preferred by the similarity‐based docking approach), (B) p38ɑ MAPK (3GC7 in green with glycine‐rich loop in blue, preferred by ALADDIN; 4KIQ_A, preferred by the similarity‐based docking approach), (C) GCR (4CSJ in green, preferred by ALADDIN; 3E7C_A in grey, preferred by the similarity‐based docking approach) and (D) CYP3A4 (4D6Z in green, preferred by ALADDIN; all others in grey).

The fact that the structure selection profile of ALADDIN differs from that of the similarity‐based docking approach can be interpreted as an indication that ALADDIN′s selection is not driven by molecular similarity between the co‐crystallized ligand and the compounds to be docked. Rather, structural characteristics of the individual protein structures are the decisive factor in structure selection (Figure 7). In the case of VEGFR2, the structure clearly preferred by ALADDIN is 1YWN. This structure is characterized by a large ligand binding pocket, which is a result of two factors: the (like in some other structures) partly unresolved DFG loop region and the co‐crystallized ligand. The co‐crystallized ligand is characterized by a distinct, bulky and rigid 5,6‐diphenylfuro[2,3‐d]pyrimidine scaffold, which contributes to a widening of the ligand binding site in particular in the region of the glycine‐rich loop). The fact that this structure obtains high early enrichment (EF_1%_=11.42; the highest value across all structures of this target) indicates that for the docking algorithm it is important to work with a widened binding pocket that allows the accommodation of the active compounds, and that the docking algorithm is able to discriminate active and inactive compounds based on protein‐ligand interaction patterns (that are only fulfilled by binders). Also the structure favored by the similarity‐based docking approach (3C7Q) has a partly unresolved DFG loop region but the observed conformation of the glycine‐rich loop leaves less space for the ligand than in 1YWN.

In the case of p38α MAPK, ALADDIN shows a preference for 3GC7, a structure bound with one of the largest co‐crystallized ligands. The ligand binding site of 3GC7 is widened compared to most other structures of p38α MAPK (including those preferred by the similarity‐based docking approach). In particular the region of the glycine‐rich loop contributes to a more open conformation of the ligand binding pocket in 3GC7 as compared to those observed in other crystal structures. Hence the conclusion that can be drawn from these observations is similar as for VEGFR2: widened binding pockets appear to be preferable for docking because they enable the algorithm to better accommodate active compounds while maintaining the ability to correctly classify inactive compounds due to a lack of compatible protein‐ligand interactions. A similar finding was obtained by Rueda et al., who noted that optimum results could be expected for protein structures with large co‐crystallized compounds (and therefore widened binding pockets).^48^

Whereas in the case of the two kinases substantial conformational changes of the protein backbone are observed, structural variations are more subtle for GCR. For GCR, important conformational changes of individual amino acids are observed, in particular for Arg611, Gln642 and Thr739. In the structures preferred by ALADDIN (and ensemble docking; 4CSJ), the orientations of the side chains of these residues allow the formation of hydrogen bonds with small molecules such as steroids. In contrast, in many of the less frequently selected structures rotamers are observed that do not allow the formation of such interactions. Also, the bulky 2,4,6‐trimethyl‐benzenesulfonamide moiety of the co‐crystallized ligand leads to a widened binding pocket.

In the case of CYP3A4, substantial conformational variability is observed across large parts of the ligand binding pocket. What distinguishes the protein structure selected by ALADDIN (4D6Z) from most other protein structures is the orientation of the side chain of Arg212, away from the ligand binding pocket. Hence, also for this protein we observe that the structure preferred by ALADDIN is one with a widened ligand binding pocket.

**Figure 8:**
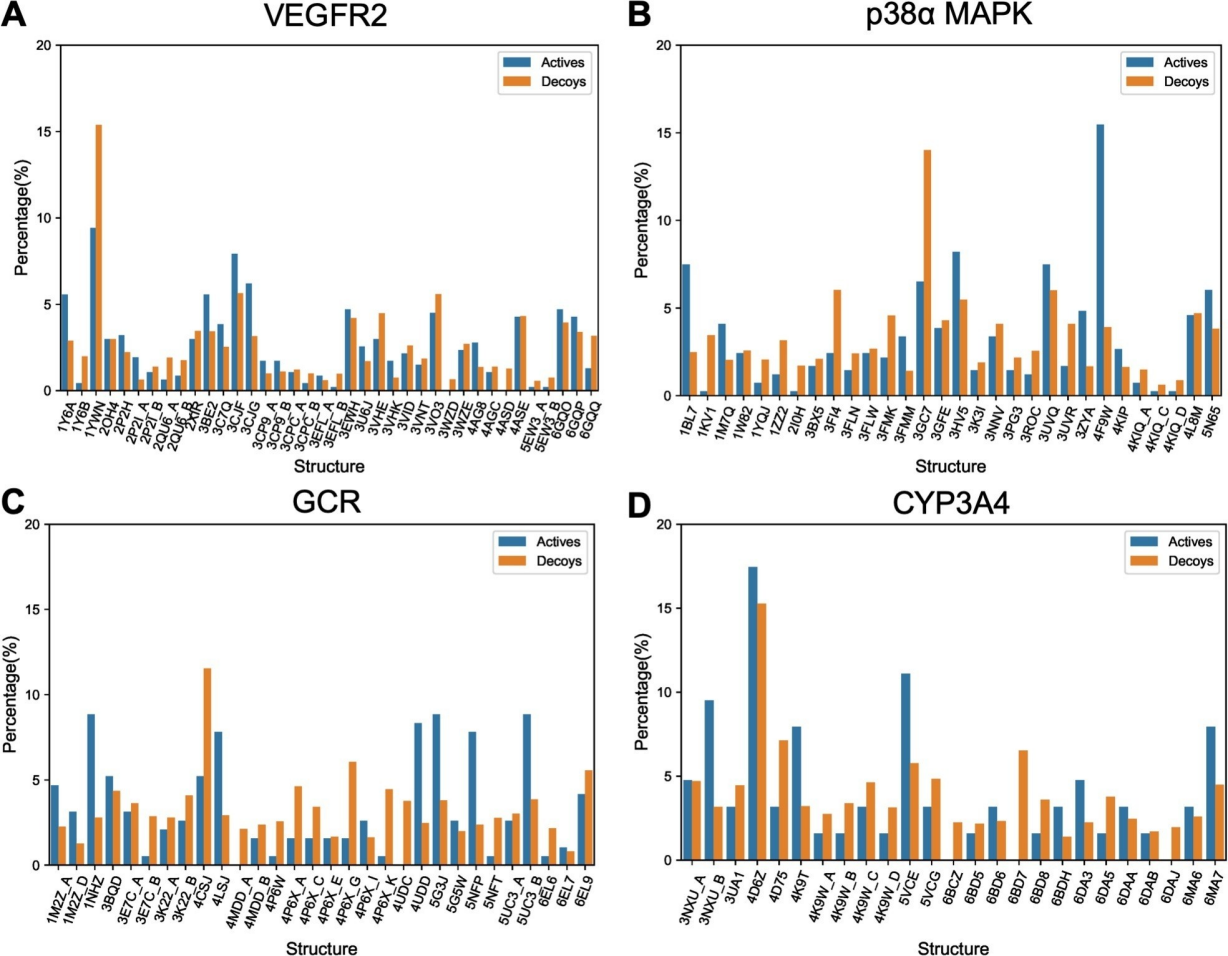
Proportion of active compounds and decoys selected by ALADDIN for docking against the individual protein structures: (A) VEGFR2, (B) p38α MAPK, (C) GCR and (D) CYP3A4.

Importantly, the proportion of active compounds and decoys selected by ALADDIN is generally well‐balanced across the individual protein structures (Figure 8). Likewise, the proportion of compounds predicted by ALADDIN as active or inactive is well‐balanced (Figure S2). These results confirm that ALADDIN does not bias structure selection in a way that, for example, active compounds are docked against “good” protein structures and decoys against “bad” ones. The classifiers do not learn to distinguish active compounds from decoys but to distinguish, as intended, compounds for which it is likely that the docking protocol will produce correct results from those for which this is less likely.

## 4 Conclusion

In this work we present ALADDIN, a new approach that integrates machine learning and docking to yield virtual screening performance superior to established docking approaches, including in particular also (all‐against‐all) ensemble docking. ALADDIN employs a battery of machine learning models to select, individually for each compound of interest, from an ensemble of protein structures, the single most suitable protein structure for docking. This makes ALADDIN not only more accurate but also faster than the established all‐against‐all ensemble docking approach as it requires any compound of interest to be docked only against a single protein structure. A further advantage of ALADDIN over existing ensemble docking approaches is that it implicitly accounts for aspects that are of major relevance to docking and scoring: protein flexibility, solvation, and the specifics of the docking algorithm and scoring function used.

ALADDIN was tested on four challenging targets. For VEGFR2, p38α MAPK, and GCR, gains in AUC over the best existing approach tested in this work were 0.05, 0.10, and 0.11, respectively. Only for CYP3A4, ALADDIN, like any of the other tested approach, did not yield decent performance. Interestingly, for kinases and GCR alike, ALADDIN preferably selected structures with a widened binding pocket, which apparently enables the docking algorithm to better accommodate active compounds while maintaining the ability to correctly identify inactive compounds.

The application of ALADDIN is limited to targets for which, as a minimum requirement, several target structures (either determined by experiment or derived by homology modeling) and a substantial number of known active compounds are available. The number of known inactive compounds may be less critical because approaches such as the DUD‐E decoys generator^34^ may be used to produce sets of putative inactive compounds.

Whereas the need for substantial amounts of biological data limits the applicability of ALADDIN, the approach can be highly useful for established, challenging targets for which there is a continued interest. These include, for example, kinases and viral proteins such as human immunodeficiency virus (HIV) type 1 protease and influenza neuraminidase. ALADDIN may also open new avenues for the development of structure‐based profilers of kinase selectivity. Importantly, ALADDIN could be highly useful for structure‐based screening of small molecules against anti‐targets.

## Abbreviations

ALADDIN: mAchine Learning AnD DockINg
AUC: area under the ROC curve
CYP3A4: Cytochrome P450 3A4
DUD‐E: directory of useful decoys, enhanced
GCR: Glucocorticoid receptor
p38α MAPK: MAP kinase p38 alpha
ROC: receiver operating characteristic
SMOTE: Synthetic Minority Over‐sampling Technique
VEGFR2: Vascular endothelial growth factor receptor 2

## Conflict of Interest

None declared.

## Supporting information

As a service to our authors and readers, this journal provides supporting information supplied by the authors. Such materials are peer reviewed and may be re‐organized for online delivery, but are not copy‐edited or typeset. Technical support issues arising from supporting information (other than missing files) should be addressed to the authors.

Supplementary
