# Supplementary material for: ALADDIN: Docking Approach Augmented by Machine Learning for Protein Structure Selection Yields Superior Virtual Screening Performance
Source: Mol Inform. 2019 Nov 8;39(4):1900103. doi: 10.1002/minf.201900103 (PMC7187304; doi:10.1002/minf.201900103)
Supplement: Supplementary file 1 — Supplementary [file MINF-39-1900103-s001.pdf]

molecular informatics

Supporting Information

ALADDIN: Docking Approach Augmented by Machine Learning for Protein Structure Selection Yields Superior Virtual Screening Performance

Ningning Fan, Christoph A. Bauer, Conrad Stork, Christina de Bruyn Kops, and Johannes Kirchmair*© 2019 The Authors. Published by Wiley-VCH Verlag GmbH & Co. KGaA. This is an open access article under the terms of the Creative Commons Attribution License, which permits use, distribution and reproduction in any medium, provided the original work is properly cited.

Supporting Information

ALADDIN: Docking Approach Augmented by Machine Learning for Protein Structure Selection Yields Superior Virtual Screening Performance

Ningning Fan,[a] Christoph A. Bauer,[b,c] Conrad Stork,[a] Christina de Bruyn Kops,[a] and Johannes Kirchmair*[a,b,c]

[a] Universität Hamburg, Faculty of Mathematics, Informatics and Natural Sciences, Department of Informatics, Center for Bioinformatics, 20146 Hamburg, Germany

[b] University of Bergen, Department of Chemistry, N-5020 Bergen, Norway

[c] University of Bergen, Computational Biology Unit (CBU), N-5020 Bergen, Norway

*e-mail: johannes.kirchmair@uib.no. Tel.: +47-55-58-34-64.

Table of Contents

Figure S1. ROC curves and AUC values obtained on the training sets of (A) VEGFR2, (B) p38α MAPK, (C) GCR and (D) CYP3A4 2

Figure S2. Proportion of compounds of the test sets predicted by ALADDIN as active or inactive for the individual protein structures. 2

Table S1. PDB Identifiers and AUC Values for All Protein Structures Selected for VEGFR2 3

Table S2. PDB Identifiers and AUC Values for All Protein Structures Selected for p38α MAPK. 3

Table S3. PDB Identifiers and AUC Values for All Protein Structures Selected for GCR. 4

Table S4. PDB Identifiers and AUC Values for All Protein Structures Selected for CYP3A4. 4

Table S5. Results of the Grid Search with 10-Fold Cross-validation for VEGFR2 4

Table S6. Results of the Grid Search with 10-Fold Cross-validation for p38α MAPK 9

Table S7. Results of the Grid Search with 10-Fold Cross-validation for GCR. 10

Table S8. Results of the Grid Search with 10-Fold Cross-validation for CYP3A4. 12

Table S9. Enrichment Factors and AUC Values for the VEGFR2 Training and Test Sets. 14

Table S10. Enrichment Factors and AUC Values for the p38α MAPK Training and Test Sets. 14

Table S11. Enrichment Factors and AUC Values for the GCR Training and Test Sets 15

Table S12. Enrichment Factors and AUC Values for the CYP3A4 Training and Test Sets. 15

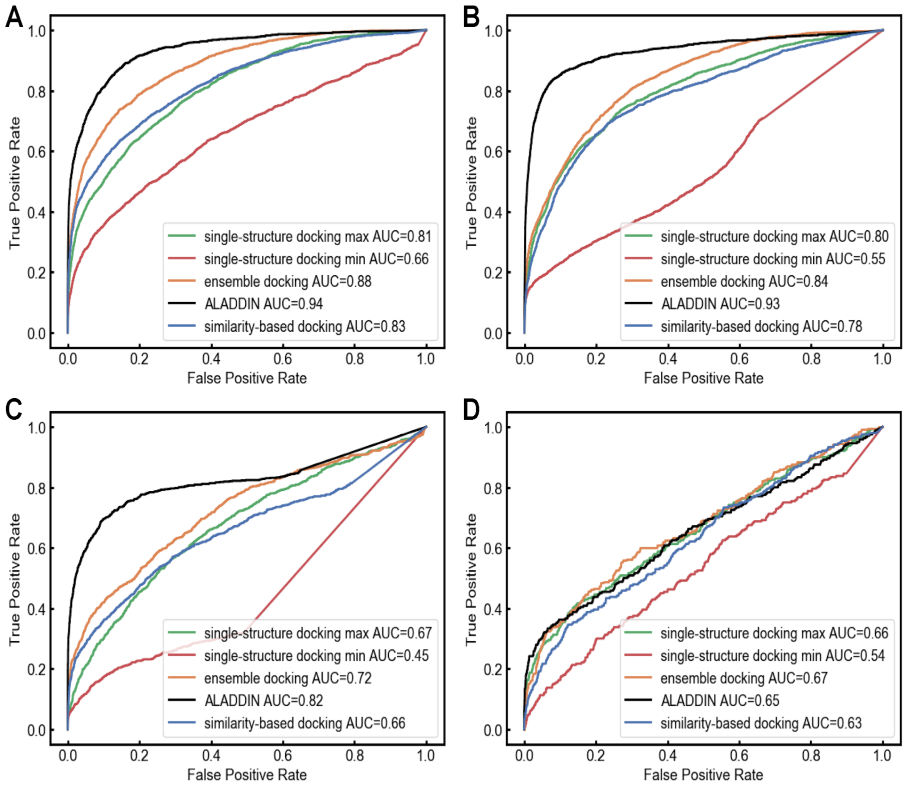

Figure S1. ROC curves and AUC values obtained on the training sets of (A) VEGFR2, (B) p38α MAPK, (C) GCR and (D) CYP3A4.

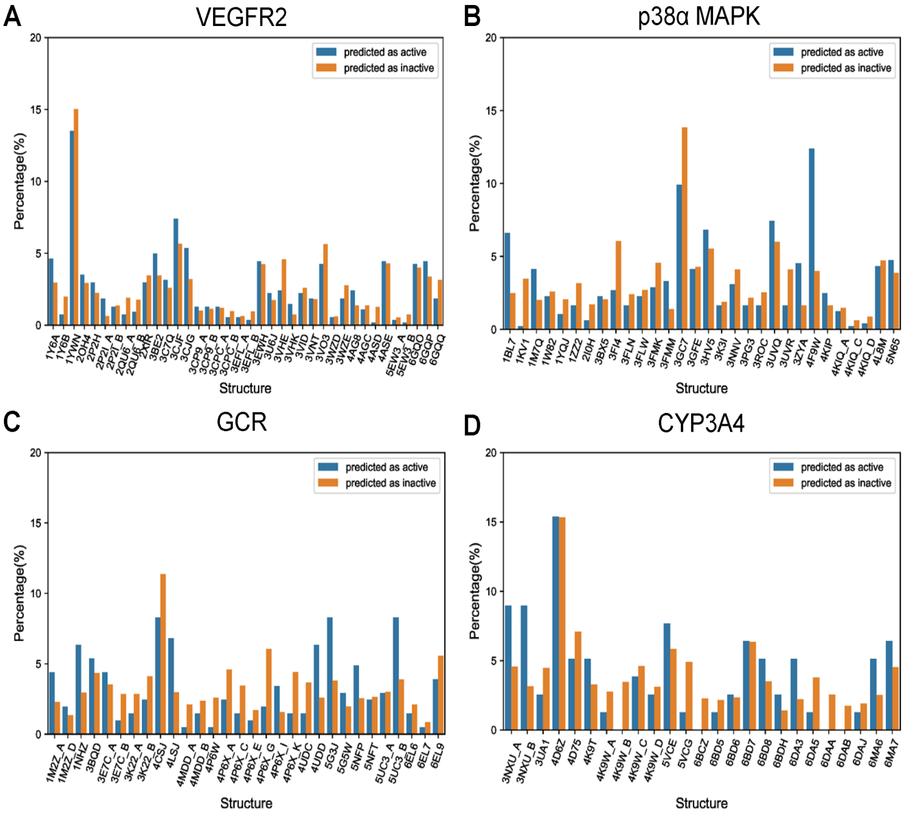

Figure S2. Proportion of compounds of the test sets predicted by ALADDIN as active or inactive for the individual protein structures: (A) VEGFR2, (B) p38α MAPK, (C) GCR and (D) CYP3A4.

Table S1. PDB Identifiers and AUC Values for All Protein Structures Selected for VEGFR2.

PDB ID	AUC[a]	PDB ID	AUC[a]	PDB ID	AUC[a]
1Y6A	0.72	3CJG	0.77	3VO3	0.81
1Y6B	0.73	3CP9_A	0.75	3WZD	0.74
1YWN	0.79	3CP9_B	0.73	3WZE	0.80
2OH4	0.76	3CPC_A	0.75	4AG8	0.79
2P2H	0.80	3CPC_B	0.72	4AGC	0.78
2P2I_A	0.71	3EFL_A	0.68	4ASD	0.77
2P2I_B	0.69	3EFL_B	0.71	4ASE	0.81
2QU6_A	0.75	3EWH	0.81	5EW3_A	0.67
2QU6_B	0.74	3U6J	0.78	5EW3_B	0.68
2XIR	0.81	3VHE	0.78	6GQO	0.81
3BE2	0.78	3VHK	0.70	6GQP[b]	0.81
3C7Q	0.73	3VID[c]	0.66	6GQQ	0.79
3CJF	0.76	3VNT	0.76		

[a] AUC values calculated for the full data set. [b] Best AUC value. [c] Worst AUC value.

Table S2. PDB Identifiers and AUC Values for All Protein Structures Selected for p38α MAPK.

PDB ID	AUC[a]	PDB ID	AUC[a]	PDB ID	AUC[a]
1BL7	0.71	3FLW	0.75	3UVQ[b]	0.80
1KV1	0.55	3FMK	0.76	3UVR	0.63
1M7Q	0.67	3FMM	0.65	3ZYA	0.58
1W82	0.60	3GC7	0.75	4F9W	0.70
1YQJ	0.72	3GFE	0.75	4KIP	0.72
1ZZ2	0.74	3HV5	0.70	4KIQ_A	0.71
2I0H	0.68	3K3I	0.66	4KIQ_C	0.66
3BX5[c]	0.55	3NNV	0.56	4KIQ_D	0.68
3FI4	0.77	3PG3	0.60	4L8M	0.58
3FLN	0.75	3ROC	0.66	5N65	0.69

[a] AUC values calculated for the full data set. [b] Best AUC value. [c] Worst AUC value.

Table S3. PDB Identifiers and AUC Values for All Protein Structures Selected for GCR.

PDB ID	AUC[a]	PDB ID	AUC[a]	PDB ID	AUC[a]
1M2Z_A	0.51	4MDD_A	0.49	4UDD	0.58
1M2Z_D	0.51	4MDD_B	0.46	5G3J	0.60
1NHZ[b]	0.68	4P6W	0.58	5G5W	0.49
3BQD	0.56	4P6X_A	0.48	5NFP	0.53
3E7C_A	0.47	4P6X_C	0.49	5NFT	0.46
3E7C_B	0.47	4P6X_E[c]	0.45	5UC3_A	0.61
3K22_A	0.54	4P6X_G	0.48	5UC3_B	0.60
3K22_B	0.56	4P6X_I	0.49	6EL6	0.49
4CSJ	0.59	4P6X_K	0.47	6EL7	0.48
4LSJ	0.53	4UDC	0.51	6EL9	0.60

[a] AUC values calculated for the full data set. [b] Best AUC value. [c] Worst AUC value.

Table S4. PDB Identifiers and AUC Values for All Protein Structures Selected for CYP3A4.

PDB ID	AUC[a]	PDB ID	AUC[a]	PDB ID	AUC[a]
3NXU_A	0.65	4K9W_D	0.62	6DA3	0.60
3NXU_B	0.62	5VCE	0.62	6DA5	0.62
3UA1	0.62	5VCG	0.64	6DAA	0.59
4D6Z[b]	0.66	6BCZ	0.64	6DAB	0.62
4D75	0.64	6BD5	0.62	6DAJ	0.59
4K9T	0.64	6BD6	0.63	6MA6[c]	0.53
4K9W_A	0.64	6BD7	0.65	6MA7	0.62
4K9W_B	0.63	6BD8	0.63		
4K9W_C	0.66	6BDH	0.62		

[a] AUC values calculated for the full data set. [b] Best AUC value. [c] Worst AUC value.

Table S5. Results of the Grid Search with 10-Fold Cross-validation for VEGFR2.

Descriptors	Fingerprint length	Number of estimators	Maximum features	Mean MCC	STDEV	AUC	$EF_{1\%}$	$EF_{5\%}$	$EF_{10\%}$
Morgan2	2048	500	sqrt	0.59	0.02	0.94	11.55	10.18	6.87
Morgan3	2048	50	sqrt	0.58	0.01	0.92	11.28	9.67	6.56
Morgan2	2048	100	sqrt	0.58	0.02	0.93	11.44	9.81	6.63
Morgan3	2048	500	sqrt	0.58	0.02	0.93	11.44	9.94	6.91
Morgan2	2048	50	sqrt	0.58	0.02	0.93	11.44	9.75	6.62
Morgan3	2048	100	sqrt	0.58	0.01	0.93	11.49	9.87	6.69
Morgan4	2048	500	sqrt	0.58	0.02	0.93	11.66	10.10	6.82
Morgan4	2048	100	sqrt	0.58	0.01	0.93	11.71	9.94	6.70

Morgan2	2048	500	0.2	0.58	0.02	0.93	11.71	10.10	6.84
Morgan3	2048	500	0.2	0.58	0.02	0.93	11.44	9.76	6.76
Morgan2	1024	100	sqrt	0.58	0.01	0.93	11.49	9.89	6.59
Morgan2	1024	50	sqrt	0.58	0.01	0.93	11.55	9.55	6.53
Morgan2	1024	500	0.2	0.58	0.02	0.93	11.66	9.97	6.88
Morgan2	1024	100	0.2	0.58	0.02	0.93	11.55	9.71	6.65
Morgan2	1024	500	sqrt	0.58	0.02	0.94	11.71	10.04	6.91
Morgan3	2048	50	0.2	0.57	0.02	0.92	11.33	9.40	6.56
Morgan3	1024	50	sqrt	0.57	0.02	0.92	11.55	9.62	6.62
Morgan4	2048	50	0.2	0.57	0.02	0.92	11.28	9.25	6.43
Morgan3	1024	50	0.2	0.57	0.02	0.92	11.66	9.52	6.44
Morgan2	1024	50	0.2	0.57	0.02	0.92	11.49	9.43	6.54
Morgan2	1024	100	0.4	0.57	0.02	0.92	11.55	9.53	6.57
Morgan3	1024	500	sqrt	0.57	0.02	0.93	11.55	9.92	6.79
Morgan4	2048	500	0.2	0.57	0.02	0.93	11.44	9.82	6.88
Morgan2	2048	100	0.2	0.57	0.02	0.93	11.39	9.87	6.75
Morgan4	2048	50	sqrt	0.57	0.01	0.93	11.6	9.57	6.58
Morgan3	2048	100	0.2	0.57	0.02	0.93	11.39	9.56	6.63
Morgan3	1024	100	sqrt	0.57	0.02	0.93	11.55	9.91	6.71
Morgan2	2048	50	0.2	0.57	0.02	0.93	11.33	9.66	6.67
Morgan4	2048	100	0.2	0.57	0.02	0.93	11.33	9.60	6.58
Morgan3	1024	500	0.2	0.57	0.02	0.93	11.60	9.80	6.69
Morgan3	1024	100	0.2	0.57	0.02	0.93	11.55	9.89	6.62
Morgan4	1024	500	sqrt	0.57	0.02	0.93	11.55	9.89	6.76
Morgan4	1024	500	0.2	0.57	0.02	0.93	11.49	9.83	6.74
Morgan2	1024	500	0.4	0.57	0.02	0.93	11.49	9.80	6.66
Morgan4	1024	100	0.2	0.56	0.02	0.92	11.60	9.70	6.66
Morgan2	2048	100	0.4	0.56	0.02	0.92	11.33	9.48	6.52
Morgan3	2048	100	0.4	0.56	0.02	0.92	11.49	9.61	6.52
Morgan4	1024	50	0.2	0.56	0.02	0.92	11.44	9.47	6.59
Morgan4	2048	100	0.4	0.56	0.02	0.92	11.44	9.60	6.56
Morgan3	1024	100	0.4	0.56	0.02	0.92	11.28	9.55	6.45
Morgan4	1024	50	sqrt	0.56	0.02	0.92	11.44	9.27	6.45
Morgan2	2048	50	0.4	0.56	0.02	0.92	11.17	9.43	6.51
Morgan3	2048	50	0.4	0.56	0.02	0.92	11.33	9.43	6.55

Morgan4	2048	50	0.4	0.56	0.02	0.92	11.39	9.48	6.48
Morgan3	1024	50	0.4	0.56	0.02	0.92	11.39	9.29	6.34
Morgan4	1024	100	0.4	0.56	0.02	0.92	11.44	9.54	6.44
Morgan2	1024	50	0.4	0.56	0.02	0.92	11.23	9.43	6.50
Morgan4	2048	500	0.4	0.56	0.02	0.93	11.49	9.87	6.74
Morgan2	2048	500	0.4	0.56	0.02	0.93	11.55	9.79	6.74
Morgan3	2048	500	0.4	0.56	0.02	0.93	11.60	9.78	6.75
Morgan4	1024	100	sqrt	0.56	0.02	0.93	11.55	9.55	6.59
Morgan3	1024	500	0.4	0.56	0.02	0.93	11.60	9.76	6.64
Morgan4	1024	500	0.4	0.56	0.02	0.93	11.49	9.77	6.69
Morgan4	2048	50	0.6	0.55	0.02	0.91	11.01	9.19	6.43
Morgan3	1024	50	0.6	0.55	0.02	0.91	11.28	9.23	6.27
Morgan4	1024	50	0.4	0.55	0.02	0.92	11.33	9.26	6.41
Morgan4	2048	500	0.6	0.55	0.02	0.92	11.39	9.62	6.56
Morgan3	1024	500	0.6	0.55	0.02	0.92	11.39	9.63	6.55
Morgan3	2048	500	0.6	0.55	0.02	0.92	11.44	9.56	6.52
Morgan3	1024	100	0.6	0.55	0.02	0.92	11.44	9.45	6.43
Morgan4	2048	100	0.6	0.55	0.02	0.92	11.33	9.43	6.57
Morgan3	2048	100	0.6	0.55	0.02	0.92	11.33	9.34	6.48
Morgan3	2048	50	0.6	0.55	0.02	0.92	11.44	9.15	6.40
Morgan4	1024	500	0.6	0.55	0.02	0.92	11.49	9.48	6.56
Morgan2	2048	500	0.6	0.55	0.02	0.92	11.55	9.53	6.63
Morgan2	2048	100	0.6	0.55	0.02	0.92	11.33	9.26	6.52
Morgan2	2048	50	0.6	0.55	0.02	0.92	11.39	9.21	6.49
Morgan4	1024	100	0.6	0.55	0.02	0.92	11.55	9.41	6.47
Morgan2	1024	500	0.6	0.55	0.02	0.92	11.39	9.53	6.59
Morgan2	1024	100	0.6	0.55	0.02	0.92	11.44	9.39	6.47
Morgan2	1024	50	0.6	0.55	0.02	0.92	11.44	9.15	6.34
Morgan4	1024	50	0.6	0.54	0.02	0.91	11.23	9.25	6.30
Morgan3	1024	500	0.8	0.53	0.02	0.91	11.49	9.37	6.33
Morgan4	2048	500	0.8	0.53	0.02	0.91	11.23	9.29	6.37
MACCS	166	500	0.2	0.53	0.02	0.91	11.44	9.17	6.39
MOE2D	n/a	500	0.4	0.53	0.02	0.92	11.49	9.98	6.69
MOE2D	n/a	100	0.4	0.53	0.02	0.92	11.60	9.74	6.63
Morgan2	1024	500	0.8	0.53	0.02	0.92	11.44	9.30	6.45

MOE2D	n/a	500	0.2	0.53	0.02	0.93	11.60	10.07	6.78
MOE2D	n/a	100	0.2	0.53	0.02	0.93	11.60	9.92	6.74
Morgan4	1024	100	0.8	0.52	0.02	0.90	11.17	9.00	6.14
Morgan4	1024	50	0.8	0.52	0.02	0.90	11.12	8.89	6.16
MACCS	166	100	sqrt	0.52	0.02	0.90	11.28	8.92	6.14
MACCS	166	50	0.2	0.52	0.02	0.90	11.06	8.61	6.12
MACCS	166	50	sqrt	0.52	0.02	0.90	11.44	8.86	6.06
MACCS	166	50	0.4	0.52	0.02	0.90	11.17	8.75	6.18
Morgan4	2048	50	0.8	0.52	0.02	0.91	11.17	8.82	6.18
Morgan3	1024	100	0.8	0.52	0.02	0.91	11.28	9.13	6.23
Morgan3	2048	500	0.8	0.52	0.02	0.91	11.12	9.28	6.39
Morgan4	2048	100	0.8	0.52	0.02	0.91	11.06	8.93	6.29
Morgan4	1024	500	0.8	0.52	0.02	0.91	11.23	9.24	6.29
Morgan3	2048	50	0.8	0.52	0.02	0.91	10.90	8.92	6.32
Morgan3	2048	100	0.8	0.52	0.02	0.91	11.33	9.04	6.35
Morgan3	1024	50	0.8	0.52	0.02	0.91	11.17	8.88	6.12
Morgan2	1024	100	0.8	0.52	0.02	0.91	11.44	9.04	6.22
MACCS	166	500	sqrt	0.52	0.02	0.91	11.33	9.20	6.41
MACCS	166	100	0.2	0.52	0.02	0.91	11.12	8.81	6.21
Morgan2	1024	50	0.8	0.52	0.02	0.91	11.23	8.82	6.11
MACCS	166	500	0.4	0.52	0.02	0.91	11.49	9.21	6.31
MACCS	166	100	0.4	0.52	0.02	0.91	11.17	9.01	6.18
Morgan2	2048	500	0.8	0.52	0.02	0.92	11.23	9.20	6.49
Morgan2	2048	50	0.8	0.52	0.02	0.92	11.28	8.87	6.40
Morgan2	2048	100	0.8	0.52	0.02	0.92	11.33	9.04	6.47
MOE2D	n/a	50	0.2	0.52	0.02	0.92	11.55	9.75	6.66
MOE2D	n/a	500	0.6	0.52	0.02	0.92	11.44	9.81	6.61
MOE2D	n/a	50	0.4	0.52	0.02	0.92	11.60	9.66	6.56
MOE2D	n/a	100	0.6	0.52	0.02	0.92	11.44	9.61	6.55
MOE2D	n/a	50	0.6	0.52	0.02	0.92	11.44	9.48	6.49
MOE2D	n/a	50	sqrt	0.52	0.02	0.92	11.44	9.65	6.52
MOE2D	n/a	500	sqrt	0.52	0.02	0.93	11.60	10.07	6.83
MOE2D	n/a	100	sqrt	0.52	0.02	0.93	11.44	9.99	6.83
MACCS	166	100	0.6	0.51	0.02	0.90	11.23	8.86	6.09
MACCS	166	50	0.6	0.51	0.02	0.90	11.01	8.76	6.15

MACCS	166	500	0.6	0.51	0.02	0.91	11.44	8.94	6.18
MOE2D	n/a	500	0.8	0.51	0.02	0.91	11.44	9.52	6.45
MOE2D	n/a	100	0.8	0.51	0.02	0.91	11.39	9.29	6.37
MOE2D	n/a	50	0.8	0.5	0.02	0.91	11.17	9.07	6.36
MACCS	166	500	0.8	0.49	0.02	0.90	11.28	8.70	6.08
MACCS	166	100	0.8	0.49	0.02	0.90	11.44	8.68	6.08
MACCS	166	50	0.8	0.49	0.02	0.90	11.12	8.58	6.13
Morgan2	2048	500	None	0.43	0.02	0.90	10.52	9.01	6.71
Morgan2	2048	50	None	0.43	0.02	0.90	10.52	9.02	6.70
Morgan2	2048	100	None	0.43	0.02	0.90	10.52	9.01	6.70
Morgan3	2048	500	None	0.42	0.02	0.90	10.36	9.00	6.50
Morgan3	2048	50	None	0.42	0.02	0.90	10.42	9.04	6.54
Morgan2	1024	500	None	0.42	0.02	0.90	10.52	8.93	6.47
Morgan2	1024	100	None	0.42	0.02	0.90	10.63	8.96	6.48
Morgan2	1024	50	None	0.42	0.02	0.90	10.69	8.98	6.49
Morgan3	2048	100	None	0.41	0.02	0.90	10.47	9.03	6.52
MOE2D	n/a	100	None	0.4	0.02	0.88	10.09	8.58	6.08
MOE2D	n/a	500	None	0.4	0.02	0.88	10.09	8.58	6.05
MOE2D	n/a	50	None	0.4	0.02	0.88	10.15	8.63	6.10
Morgan4	2048	500	None	0.4	0.02	0.89	10.52	8.79	6.42
Morgan4	2048	100	None	0.4	0.02	0.89	10.47	8.76	6.42
Morgan4	2048	50	None	0.4	0.02	0.89	10.42	8.77	6.42
Morgan3	1024	500	None	0.4	0.02	0.89	10.36	8.84	6.41
Morgan3	1024	100	None	0.4	0.02	0.89	10.31	8.87	6.42
Morgan3	1024	50	None	0.4	0.02	0.89	10.36	8.84	6.41
MACCS	166	50	None	0.4	0.02	0.89	10.31	8.70	6.23
MACCS	166	100	None	0.4	0.02	0.89	10.31	8.72	6.25
MACCS	166	500	None	0.4	0.02	0.89	10.31	8.72	6.25
Morgan4	1024	500	None	0.38	0.02	0.89	10.74	8.90	6.40
Morgan4	1024	50	None	0.38	0.02	0.89	10.69	8.85	6.40
Morgan4	1024	100	None	0.38	0.02	0.89	10.69	8.89	6.42

Table S6. Results of the Grid Search with 10-Fold Cross-validation for p38α MAPK.

Descriptors	Fingerprint length	Number of estimators	Maximum features	Mean MCC	STDEV	AUC	EF1%	EF5%	EF10%
Morgan2	1024	500	sqrt	0.60	0.03	0.93	16.08	11.88	7.95
Morgan2	1024	100	sqrt	0.60	0.03	0.93	16.02	11.45	7.82
Morgan2	1024	50	sqrt	0.60	0.03	0.93	15.24	10.99	7.65
Morgan2	1024	500	0.2	0.59	0.03	0.93	15.69	11.53	7.68
Morgan2	1024	100	0.2	0.59	0.03	0.92	15.52	11.01	7.52
Morgan2	1024	50	0.2	0.59	0.03	0.92	15.35	10.68	7.51
Morgan2	1024	500	0.4	0.59	0.03	0.92	15.58	11.35	7.52
Morgan2	1024	100	0.4	0.59	0.03	0.92	15.63	10.79	7.45
Morgan2	1024	50	0.4	0.58	0.03	0.92	15.35	10.39	7.39
Morgan2	1024	500	0.6	0.58	0.03	0.91	15.63	10.98	7.37
Morgan2	1024	100	0.6	0.57	0.03	0.91	14.91	10.55	7.28
Morgan2	1024	50	0.6	0.57	0.03	0.91	14.69	10.19	7.21
Morgan2	1024	50	0.8	0.55	0.03	0.90	13.91	9.55	6.91
Morgan2	1024	100	0.8	0.55	0.03	0.90	14.63	9.84	6.94
Morgan2	1024	500	0.8	0.55	0.03	0.90	14.86	10.41	7.01
MACCS	166	500	sqrt	0.55	0.03	0.91	15.52	10.81	7.29
MOE2D	n/a	500	0.2	0.54	0.03	0.92	16.08	11.44	7.52
MACCS	166	500	0.2	0.54	0.03	0.91	15.63	10.82	7.36
MACCS	166	100	sqrt	0.54	0.03	0.91	15.24	10.51	7.33
MOE2D	n/a	100	0.2	0.54	0.03	0.92	15.69	10.90	7.49
MACCS	166	100	0.2	0.54	0.03	0.91	15.19	10.52	7.20
MACCS	166	50	sqrt	0.54	0.03	0.90	14.63	9.98	7.10
MOE2D	n/a	500	0.4	0.54	0.03	0.91	16.02	11.04	7.38
MACCS	166	50	0.2	0.54	0.03	0.91	14.80	10.00	7.09
MOE2D	n/a	50	0.2	0.54	0.03	0.91	15.19	10.44	7.31
MOE2D	n/a	100	0.4	0.54	0.03	0.91	15.63	10.59	7.22
MACCS	166	500	0.4	0.54	0.03	0.91	15.47	10.80	7.28
MOE2D	n/a	500	sqrt	0.54	0.03	0.93	15.74	11.71	7.67
MACCS	166	100	0.4	0.54	0.03	0.90	15.52	10.32	7.06
MOE2D	n/a	50	0.4	0.54	0.03	0.91	15.74	10.21	7.14
MACCS	166	50	0.4	0.54	0.03	0.90	14.75	9.86	7.00
MOE2D	n/a	100	sqrt	0.54	0.03	0.92	15.69	10.95	7.44
MOE2D	n/a	500	0.6	0.54	0.03	0.91	15.74	10.80	7.24

MOE2D	n/a	50	sqrt	0.53	0.03	0.91	15.30	10.53	7.37
MACCS	166	500	0.6	0.53	0.03	0.90	14.86	10.50	7.08
MOE2D	n/a	100	0.6	0.53	0.03	0.91	15.47	10.38	7.17
MACCS	166	100	0.6	0.53	0.03	0.90	14.58	10.02	6.92
MACCS	166	50	0.6	0.53	0.03	0.89	14.30	9.59	6.88
MOE2D	n/a	50	0.6	0.53	0.03	0.90	15.02	9.81	6.98
MOE2D	n/a	500	0.8	0.52	0.03	0.90	15.41	10.19	6.92
MOE2D	n/a	100	0.8	0.52	0.03	0.89	14.75	9.63	6.77
MACCS	166	500	0.8	0.52	0.03	0.90	14.69	10.30	7.06
MOE2D	n/a	50	0.8	0.51	0.03	0.89	14.41	9.76	6.92
MACCS	166	100	0.8	0.51	0.03	0.90	14.19	9.65	6.89
MACCS	166	50	0.8	0.51	0.03	0.89	13.86	9.30	6.86
Morgan2	1024	500	None	0.42	0.04	0.87	13.75	9.14	6.84
Morgan2	1024	50	None	0.42	0.04	0.88	13.91	9.30	6.89
Morgan2	1024	100	None	0.42	0.04	0.87	13.69	9.24	6.88
MACCS	166	500	None	0.40	0.03	0.86	14.08	9.59	6.80
MACCS	166	50	None	0.40	0.03	0.86	13.97	9.57	6.79
MACCS	166	100	None	0.40	0.03	0.86	13.97	9.59	6.80
MOE2D	n/a	500	None	0.38	0.03	0.85	12.03	8.78	6.35
MOE2D	n/a	100	None	0.38	0.03	0.85	12.20	8.81	6.33
MOE2D	n/a	50	None	0.38	0.03	0.85	12.14	8.86	6.36

Table S7. Results of the Grid Search with 10-Fold Cross-validation for GCR.

Descriptors	Fingerprint length	Number of estimators	Maximum features	Mean MCC	STDEV	AUC	$EF_{1\%}$	$EF_{5\%}$	$EF_{10\%}$
Morgan2	1024	500	sqrt	0.62	0.02	0.82	15.75	9.90	6.30
Morgan2	1024	100	sqrt	0.62	0.02	0.80	15.50	9.28	5.96
Morgan2	1024	50	sqrt	0.62	0.02	0.79	15.13	8.58	5.61
Morgan2	1024	500	0.2	0.61	0.02	0.80	15.38	9.35	6.06
Morgan2	1024	100	0.2	0.61	0.02	0.79	15.00	8.88	5.79
Morgan2	1024	50	0.2	0.61	0.02	0.77	15.25	8.38	5.38
Morgan2	1024	500	0.4	0.61	0.02	0.79	15.25	9.30	5.96
Morgan2	1024	100	0.4	0.61	0.02	0.77	15.25	8.48	5.50
Morgan2	1024	50	0.4	0.60	0.02	0.76	15.00	8.30	5.28
Morgan2	1024	500	0.6	0.59	0.02	0.77	15.13	8.70	5.64
MACCS	166	500	sqrt	0.59	0.02	0.79	14.63	8.78	5.69

Morgan2	1024	50	0.6	0.59	0.02	0.74	15.13	8.05	5.04
MACCS	166	100	sqrt	0.59	0.02	0.77	14.63	8.03	5.20
Morgan2	1024	100	0.6	0.59	0.02	0.76	15.38	8.20	5.38
MOE2D	n/a	500	sqrt	0.59	0.02	0.82	15.63	10.38	6.38
MACCS	166	50	sqrt	0.59	0.02	0.75	14.75	7.85	5.01
MACCS	166	500	0.2	0.59	0.02	0.78	14.50	8.48	5.46
MOE2D	n/a	500	0.2	0.59	0.02	0.82	15.75	10.10	6.30
MACCS	166	100	0.2	0.59	0.02	0.75	14.75	7.95	5.11
MOE2D	n/a	100	0.2	0.59	0.02	0.80	15.50	9.45	5.95
MOE2D	n/a	100	sqrt	0.59	0.02	0.81	15.63	9.60	6.00
MACCS	166	50	0.2	0.59	0.02	0.74	14.38	7.83	4.98
MOE2D	n/a	50	sqrt	0.59	0.02	0.80	15.50	9.25	5.84
MOE2D	n/a	50	0.2	0.58	0.02	0.78	15.25	8.85	5.50
MOE2D	n/a	500	0.4	0.58	0.02	0.81	15.63	9.83	6.20
MACCS	166	500	0.4	0.58	0.02	0.77	14.75	8.48	5.45
MACCS	166	100	0.4	0.58	0.02	0.76	14.75	7.93	5.20
MOE2D	n/a	100	0.4	0.58	0.02	0.80	15.50	9.45	5.85
MOE2D	n/a	50	0.4	0.58	0.02	0.77	14.63	8.68	5.55
MACCS	166	50	0.4	0.58	0.02	0.73	14.38	7.55	4.91
MOE2D	n/a	500	0.6	0.58	0.02	0.79	15.63	9.35	5.88
MACCS	166	100	0.6	0.58	0.02	0.74	14.38	7.70	5.00
MACCS	166	500	0.6	0.58	0.02	0.76	14.75	8.30	5.26
MACCS	166	50	0.6	0.58	0.02	0.73	14.63	7.33	4.85
MOE2D	n/a	100	0.6	0.58	0.02	0.77	15.25	8.70	5.55
MOE2D	n/a	50	0.6	0.57	0.02	0.76	15.38	8.30	5.26
MACCS	166	500	0.8	0.56	0.02	0.75	14.38	7.95	5.00
Morgan2	1024	500	0.8	0.56	0.03	0.75	15.00	8.30	5.28
MACCS	166	100	0.8	0.56	0.02	0.73	14.38	7.43	4.76
MACCS	166	50	0.8	0.56	0.02	0.71	13.88	6.98	4.58
Morgan2	1024	50	0.8	0.56	0.03	0.71	14.63	7.43	4.65
Morgan2	1024	100	0.8	0.56	0.03	0.73	14.63	7.83	4.89
MOE2D	n/a	500	0.8	0.56	0.02	0.77	15.38	8.95	5.63
MOE2D	n/a	100	0.8	0.55	0.02	0.75	15.13	8.23	5.19
MOE2D	n/a	50	0.8	0.55	0.02	0.74	14.38	7.83	5.03
MACCS	166	500	None	0.47	0.02	0.68	12.63	5.88	3.90

MACCS	166	50	None	0.47	0.02	0.68	12.63	5.88	3.90
MACCS	166	100	None	0.47	0.02	0.68	12.63	5.88	3.90
Morgan2	1024	100	None	0.45	0.02	0.66	12.63	5.88	3.89
Morgan2	1024	500	None	0.45	0.02	0.66	12.75	5.85	3.85
Morgan2	1024	50	None	0.45	0.02	0.66	12.63	5.90	3.93
MOE2D	n/a	500	None	0.45	0.02	0.67	12.63	6.00	3.88
MOE2D	n/a	50	None	0.45	0.02	0.67	12.75	6.00	3.91
MOE2D	n/a	100	None	0.45	0.02	0.67	12.63	5.98	3.89

Table S8. Results of the Grid Search with 10-Fold Cross-validation for CYP3A4.

Descriptors	Fingerprint length	Number of estimators	Maximum features	Mean MCC	STDEV	AUC	$EF_{1\%}$	$EF_{5\%}$	$EF_{10\%}$
Morgan2	1024	500	sqrt	0.50	0.02	0.65	18.33	5.92	3.54
Morgan2	1024	500	0.2	0.50	0.02	0.65	17.92	5.83	3.54
Morgan2	1024	100	sqrt	0.50	0.02	0.62	17.08	5.33	3.17
Morgan2	1024	100	0.2	0.50	0.02	0.63	17.50	5.92	3.33
Morgan2	1024	50	sqrt	0.50	0.02	0.64	17.50	5.75	3.46
Morgan2	1024	50	0.2	0.50	0.02	0.63	18.75	6.00	3.38
Morgan2	1024	500	0.4	0.49	0.02	0.63	15.83	5.33	3.33
Morgan2	1024	100	0.4	0.48	0.03	0.62	16.25	5.92	3.50
Morgan2	1024	50	0.4	0.48	0.02	0.63	17.92	5.92	3.42
Morgan2	1024	500	0.6	0.47	0.02	0.64	17.08	5.67	3.46
Morgan2	1024	100	0.6	0.47	0.02	0.63	17.50	5.75	3.25
Morgan2	1024	50	0.6	0.47	0.02	0.63	18.33	5.58	3.25
MACCS	166	500	0.2	0.46	0.02	0.64	14.58	5.42	3.42
MACCS	166	100	0.2	0.46	0.02	0.62	15.00	5.08	3.25
MACCS	166	500	sqrt	0.46	0.02	0.62	15.42	5.25	3.33
MACCS	166	100	sqrt	0.46	0.02	0.63	15.83	5.00	3.25
MACCS	166	500	0.4	0.46	0.02	0.62	13.75	4.92	3.17
MACCS	166	50	0.2	0.45	0.02	0.61	15.00	5.42	3.46
MACCS	166	50	sqrt	0.45	0.02	0.62	16.67	5.17	3.33
MACCS	166	100	0.4	0.45	0.02	0.63	13.75	5.42	3.29
MACCS	166	50	0.4	0.45	0.02	0.63	15.83	5.58	3.29
MACCS	166	500	0.6	0.44	0.02	0.62	13.33	5.00	3.08
MACCS	166	100	0.6	0.44	0.02	0.63	14.58	5.17	3.46
MACCS	166	50	0.6	0.44	0.02	0.63	16.25	5.33	3.38

Morgan2	1024	500	0.8	0.43	0.02	0.64	15.42	5.17	3.33
Morgan2	1024	50	0.8	0.43	0.02	0.63	18.33	5.75	3.46
Morgan2	1024	100	0.8	0.42	0.02	0.64	17.08	5.58	3.42
MACCS	166	500	0.8	0.41	0.02	0.62	16.25	5.33	3.25
MACCS	166	100	0.8	0.41	0.02	0.61	15.42	5.17	3.29
MACCS	166	50	0.8	0.41	0.02	0.62	16.25	5.33	3.33
MOE2D	n/a	500	0.2	0.39	0.02	0.66	17.50	5.92	3.67
MOE2D	n/a	100	0.2	0.39	0.02	0.63	14.58	5.17	3.33
MOE2D	n/a	500	0.4	0.39	0.02	0.66	17.50	6.00	3.71
MOE2D	n/a	50	0.2	0.39	0.02	0.66	18.75	5.83	3.63
MOE2D	n/a	100	0.4	0.39	0.02	0.65	16.25	5.75	3.54
MOE2D	n/a	50	0.4	0.38	0.02	0.65	16.67	5.50	3.50
MOE2D	n/a	100	sqrt	0.38	0.02	0.65	17.92	5.75	3.50
MOE2D	n/a	500	sqrt	0.38	0.02	0.66	17.92	5.83	3.63
MOE2D	n/a	500	0.6	0.38	0.02	0.65	16.67	5.42	3.50
MOE2D	n/a	100	0.6	0.38	0.02	0.65	15.42	5.25	3.29
MOE2D	n/a	50	sqrt	0.38	0.02	0.62	16.67	5.17	3.21
MOE2D	n/a	50	0.6	0.37	0.02	0.64	16.25	5.33	3.54
MOE2D	n/a	500	0.8	0.35	0.02	0.62	14.17	5.00	3.33
MOE2D	n/a	50	0.8	0.34	0.02	0.63	15.00	5.25	3.29
MOE2D	n/a	100	0.8	0.34	0.02	0.62	13.33	4.92	3.29
MACCS	166	100	None	0.31	0.02	0.62	15.83	5.42	3.25
MACCS	166	50	None	0.31	0.02	0.62	15.83	5.42	3.25
MACCS	166	500	None	0.31	0.02	0.62	15.83	5.42	3.25
Morgan2	1024	500	None	0.30	0.02	0.65	17.50	5.25	3.46
Morgan2	1024	50	None	0.30	0.02	0.65	17.50	5.17	3.42
Morgan2	1024	100	None	0.30	0.02	0.65	17.50	5.25	3.38
MOE2D	n/a	100	None	0.26	0.03	0.63	15.00	4.92	3.42
MOE2D	n/a	500	None	0.26	0.03	0.63	15.42	5.00	3.42
MOE2D	n/a	50	None	0.26	0.03	0.63	15.00	4.92	3.42

Table S9. Enrichment Factors and AUC Values for the VEGFR2 Training and Test Sets.

Method	Training set				Test set 1				Test subset 1				Test subset 2			
	AUC	EF			AUC	EF			AUC	EF			AUC	EF		
		1%	5%	10%		1%	5%	10%		1%	5%	10%		1%	5%	10%
Single-structure docking max	0.81	10.58	6.64	4.46	0.82	10.49	6.94	4.90	0.82	15.11	7.47	4.98	0.81	13.24	7.65	4.56
Single-structure docking min	0.66	8.90	4.68	3.28	0.66	7.49	4.33	3.00	0.68	7.11	4.71	3.24	0.65	7.35	4.12	3.09
Ensemble docking	0.88	11.49	8.00	5.77	0.88	11.56	8.27	5.70	0.87	16.89	9.24	5.64	0.84	29.41	9.71	6.03
ALADDIN	0.94	11.71	10.04	6.91	0.93	11.35	10.45	6.92	0.91	17.33	11.11	6.62	0.86	33.82	10.88	6.91
Similarity-based docking	0.83	11.44	7.66	5.06	0.83	11.35	7.92	5.01	0.82	17.33	8.36	5.07	0.83	26.47	9.71	5.44

Table S10. Enrichment Factors and AUC Values for the p38α MAPK Training and Test Sets.

Method	Training set				Test set 1				Test subset 1				Test subset 2			
	AUC	EF			AUC	EF			AUC	EF			AUC	EF		
		1%	5%	10%		1%	5%	10%		1%	5%	10%		1%	5%	10%
Single-structure docking max	0.80	12.58	6.60	4.71	0.79	12.32	6.91	4.61	0.79	13.54	6.99	4.67	0.79	12.00	7.20	4.80
Single-structure docking min	0.55	11.09	3.64	2.24	0.54	9.66	3.38	2.05	0.55	8.73	3.41	2.27	0.55	13.33	4.00	2.27
Ensemble docking	0.84	14.36	7.05	4.74	0.84	16.43	8.02	4.93	0.82	16.16	7.60	4.80	0.78	20.00	6.67	4.53
ALADDIN	0.93	16.08	11.88	7.95	0.94	18.12	12.51	8.14	0.94	23.58	13.45	8.30	0.91	34.67	14.40	7.87
Similarity-based docking	0.78	11.70	5.91	4.30	0.79	12.08	5.65	4.06	0.76	10.04	5.24	3.67	0.73	9.33	4.80	3.20

Table S11. Enrichment Factors and AUC Values for the GCR Training and Test Sets.

Method	Training set				Test set 1				Test subset 1				Test subset 2			
	AUC	EF			AUC	EF			AUC	EF			AUC	EF		
		1%	5%	10%		1%	5%	10%		1%	5%	10%		1%	5%	10%
Single-structure docking max	0.67	6.38	3.80	2.84	0.68	5.73	3.65	2.92	0.70	6.49	3.90	3.51	0.75	4.76	3.81	4.29
Single-structure docking min	0.45	5.63	2.28	1.68	0.46	5.73	2.81	2.03	0.55	10.39	4.68	3.25	0.69	23.81	7.62	5.71
Ensemble docking	0.72	12.00	5.80	3.96	0.71	11.46	5.21	3.39	0.72	9.09	3.38	2.47	0.71	4.76	2.86	1.90
ALADDIN	0.82	15.75	9.90	6.30	0.82	16.15	9.69	6.56	0.85	20.78	11.43	6.88	0.88	28.57	11.43	7.62
Similarity-based docking	0.66	11.00	5.23	3.35	0.66	8.85	4.69	3.02	0.69	7.79	3.64	2.60	0.61	4.76	1.90	1.43

Table S12. Enrichment Factors and AUC Values for the CYP3A4 Training and Test Sets.

Method	Training set				Test set 1				Test subset 1				Test subset 2			
	AUC	EF			AUC	EF			AUC	EF			AUC	EF		
		1%	5%	10%		1%	5%	10%		1%	5%	10%		1%	5%	10%
Single-structure docking max	0.66	15.00	5.25	3.25	0.68	19.05	5.71	3.97	0.63	17.14	4.00	3.71	0.65	26.32	6.32	4.74
Single-structure docking min	0.54	4.58	2.25	1.63	0.53	1.59	2.22	1.90	0.47	2.86	2.29	1.43	0.41	5.26	3.16	1.58
Ensemble docking	0.67	12.92	5.25	3.42	0.67	19.05	6.67	3.97	0.62	17.14	6.86	3.71	0.66	26.32	9.47	4.74
ALADDIN	0.65	18.33	5.92	3.54	0.65	20.63	7.30	3.97	0.61	20.00	7.43	3.71	0.69	31.58	9.47	4.74
Similarity-based docking	0.63	7.92	3.92	2.83	0.64	12.70	5.71	3.17	0.60	11.43	5.14	2.57	0.63	21.05	6.32	3.16
